# Hepatocyte and keratinocyte growth factors and their receptors in human lung emphysema

**DOI:** 10.1186/1471-2466-5-13

**Published:** 2005-10-10

**Authors:** Marcel Bonay, Anne Boutten, Véronique Leçon-Malas, Joëlle Marchal, Paul Soler, Michel Fournier, Guy Leseche, Monique Dehoux, Bruno Crestani

**Affiliations:** 1 INSERM U 700, Faculté Xavier Bichat, Paris, France; 2 Service de Physiologie-Explorations Fonctionnelles, Hôpital Bichat-Claude Bernard, Assistance Publique-Hôpitaux de Paris, Université Paris 7, Paris, France; 3 Service de Biochimie A, Hôpital Bichat-Claude Bernard, Assistance Publique-Hôpitaux de Paris, Université Paris 7, Paris, France; 4 Service de Pneumologie Hôpital Bichat-Claude Bernard, Assistance Publique-Hôpitaux de Paris, Université Paris 7, Paris, France; 5 Service de Pneumologie, Hôpital Beaujon, Assistance Publique-Hôpitaux de Paris, Université Paris 7, Paris, France; 6 Service de Chirurgie Thoracique, Hôpital Beaujon, Assistance Publique-Hôpitaux de Paris, Université Paris 7, Paris, France

## Abstract

**Background:**

Hepatocyte and keratinocyte growth factors are key growth factors in the process of alveolar repair. We hypothesized that excessive alveolar destruction observed in lung emphysema involves impaired expression of hepatocyte and keratinocyte growth factors or their respective receptors, c-met and keratinocyte growth factor receptor. The aim of our study was to compare the expression of hepatocyte and keratinocyte growth factors and their receptors in lung samples from 3 groups of patients: emphysema; smokers without emphysema and non-smokers without emphysema.

**Methods:**

Hepatocyte and keratinocyte growth factor proteins were analysed by immunoassay and western blot; mRNA expression was measured by real time quantitative polymerase chain reaction.

**Results:**

Hepatocyte and keratinocyte growth factors, c-met and keratinocyte growth factor receptor mRNA levels were similar in emphysema and non-emphysema patients. Hepatocyte growth factor mRNA correlated negatively with FEV1 and the FEV1/FVC ratio both in emphysema patients and in smokers with or without emphysema. Hepatocyte and keratinocyte growth factor protein concentrations were similar in all patients' groups.

**Conclusion:**

The expression of hepatocyte and keratinocyte growth factors and their receptors is preserved in patients with lung emphysema as compared to patients without emphysema. Hepatocyte growth factor mRNA correlates with the severity of airflow obstruction in smokers.

## Background

Emphysema is one of the most important cause of respiratory insufficiency with increasing mortality and morbidity [1]. Lung emphysema is defined pathologically by the destruction of alveolar walls with abnormal permanent enlargement of the air spaces distal to the terminal bronchiole. Precise mechanims contributing to the destruction of alveolar walls remain unclear. Cigarette smoking may induce emphysema by stimulating neutrophils and alveolar macrophages to produce proteases leading to the degradation of the alveolar extracellular matrix and oxidant injury contributing to alveolar destruction [2]. T lymphocytes contribute to the recruitment and activation of these inflammatory cells and may be involved in the apoptosis and destruction of alveolar epithelium [3-5]. Recent evidence of increased apoptosis of alveolar epithelial and endothelial cells in emphysematous lung suggests that primary alterations of the alveolar epithelium and endothelium might participate in the pathogenesis of the disease [6-8].

The alveolar epithelium is essential for maintenance of the integrity of the alveolar spaces. Functional restoration of the alveolar epithelium after an injury requires the proliferation and migration of type 2 pneumocytes and their differentiation into type 1 pneumocytes, a tightly regulated phenomenon. Growth factors have been shown to control both phases of the process [9-12]. Among these factors, the hepatocyte growth factor (HGF), a heterodimeric protein obtained through the cleavage of an inactive precursor, called 90-kD proHGF, and the keratinocyte growth factor (KGF, also named fibroblast growth factor-7, FGF-7) have been identified as key factors in the process of alveolar repair, both in acute or chronic conditions [13-15]. HGF and KGF respectively act through specific receptors c-met, a membrane bound tyrosine kinase [16] and FGFR2IIIb, also named KGF-R [17]. HGF and KGF productions by lung fibroblasts from emphysema have been shown to be reduced when compared with controls [18]. Recently, Shigemura et al reported that decreased HGF expression due to a failure in sustained endogenous production after injury was associated with emphysema-related histopathologic and physiological changes in a rat model of elastase-induced emphysema [19]. In this animal model, HGF could have a therapeutic effect [20,19].

We hypothesized that a defective expression of growth factors involved in human alveolar epithelium repair such as HGF and KGF or their specific receptors might participate in the pathophysiology of lung emphysema. We therefore evaluated the expression of HGF, KGF, and their receptors c-met and KGF-R in lung biopsies from patients with emphysema and from non-emphysema patients, according to their smoking status.

## Methods

This study was approved by the ethics committee of Saint Germain-en-Laye hospital.

### Patients

Lung samples were obtained during surgery in adult patients (>18 years) from Beaujon university hospital (Clichy, France) and from Bichat university hospital (Paris, France).

#### Patients with lung emphysema

Seventeen patients with radiographically defined emphysema (E group) were included. All patients were active or ex-smokers (Table 1). CT-scan, pulmonary function tests and α1-AT deficiency were systematically documented. Patients with α1-AT deficiency were excluded. They underwent bullectomy (n = 3), lobectomy (n = 4), lung transplantation (n = 3) or lung volume reduction (n = 7). Pulmonary function tests demonstrated mild to severe airflow obstruction and lung distension (Table 2). Tissue samples were taken from the resected parenchyma in a macroscopically emphysematous region. Nine patients with emphysema were receiving corticosteroids, either oral (n = 2) and/or inhaled (n = 8). In all patients, lung emphysema was suspected on CT-scan and confirmed by the pathological examination of lung resection samples. The severity of emphysema was approached through pulmonary function abnormalities.

**Table 1:** Clinical characteristics of patients with and without emphysema

	Emphysema (E)	Non-emphysema Smokers (S)	Non-emphysema Non-Smokers (NS)	Between groups differences
n	17	8	10	
Age (yr)	60 ± 7.4	55.8 ± 11.2	52.7 ± 19.4	ns
Sex ratio F/M	1/16	0/8	2/8	ns
Smoking (Pack.yr)	51 ± 21.4*	33.3 ± 14.8	0	p < 0.001
Time since smoking cessation (yearrs)	7.0 ± 7.1	10 (n = 1)	Non relevant	Non relevant
Active smokers/ex-smokers	7/10	7/1	0/0	p < 0.001

*: vs S, p < 0.05; as assessed by Mann-Whitney U-test. ns: non significant.

**Table 2:** Pulmonary function tests of patients with and without emphysema.

	Emphysema (E)	Non-emphysema Smokers (S)	Non-emphysema Non-Smokers (NS)	Between groups differences
n	17	8	10	
FEV_1 _% predicted value (% pred)	41.7 ± 26.6 *,†	71 ± 21.3	82 ± 11.1	p < 0.001
FEV_1_/FVC	48.7 ± 13.9 *,†	67.6 ± 14.8†	86.9 ± 11.6	p < 0.001
RV (%pred)	211.6 ± 69.4 *,†	123 ± 19.7	88 ± 23.9	p < 0.001
TLC (%pred)	121.5 ± 17.8 *,†	100 ± 10.4 †	83 ± 13.5	p < 0.001
Pa_O2 _(mmHg)	69.7 ± 10.1 *,†	81 ± 15.8	85.2 ± 7.6	p < 0.005
Pa_CO2 _(mmHg)	41.3 ± 5	38.7 ± 7.7	41.8 ± 2.6	ns

*: vs S, p < 0.01; †: vs NS, p < 0.01; as assessed by Mann-Whitney U-test. ns: non significant

Pa_O2_: arterial oxygen pressure, Pa_CO2_: arterial carbon dioxide pressure, FEV_1_: forced expiratory volume in one second, RV: residual volume, FVC: forced vital capacity, TLC: total lung capacity.

#### Non-emphysema patients

Normal tissue was obtained from 18 non-emphysema patients. Eight patients were smokers (non-emphysema smoker, S) and 10 were non-smokers (non-emphysema non-smoker, NS). S patients were undergoing surgery for the resection of a localised primary lung carcinoma (n = 7) or a benign lesion (n = 1). NS patients were undergoing surgery for the resection of a localised primary lung carcinoma (n = 3), lung metastases (n = 2) or a benign lesion (n = 5). Tissue samples were taken at a site distant from the pathological process and without macroscopical and microscopical evidence of emphysema. S patients had mild to moderate alterations of pulmonary functions tests (table 2). Increased cumulative tobacco exposure was observed in the E group as compared with the S group (Table 1). No difference was observed between groups for age and sex ratio (Table 1). Two patients without emphysema (S group) received inhaled corticosteroids.

### Processing of lung samples

Lung tissue fragments (about 0.2 cm^3^) were immediately frozen in liquid N_2 _and stored at -80°C until RNA and protein analysis. The histopathology of biopsies was evaluated on paraffin-embedded sections to verify the features of emphysema or normal lungs. The concentration of proteins in biopsies was evaluated from 100 mg of lung samples homogenised with 0.5 ml PBS containing 200 μM phenylmethylsulfonyl fluoride, 1 mg/ml leupeptin and 1 mg/ml aprotinin. The homogenates were centrifuged at 10,000 g for 10 min at 4°C to remove tissue fragments and the supernatants were collected and stored at -80°C until measurement.

### Quantitative analysis of mRNA expression

Total RNA was extracted from frozen lung tissue and reverse transcribed. Each sample was analysed by reverse transcriptase-real-time polymerase chain reaction (RT-PCR) with specific primers (table 3) to quantify the expression of mRNA of HGF, KGF, c-met and KGF-R as described previously [21].

**Table 3:** Primers and PCR cycling conditions.

Primers	Sequences	Denaturation	annealing	Cycles	PCR products
HGF: Forward Reverse	5'-CAGAGGGACAAAGGAAAAGAA-3' 5'-GCAAGTGAATGGAAGTCCTTTA-3'	94°C, 15s	58°C, 60s	40	167 bp
KGF: Forward Reverse	5'-GAACAAGGAAGGAAAACTCTATGCAA-3' 5'-AAGTGGGCTGTTTTTTGTTCTTTCT-3'	94°C, 15s	60°C, 60s	40	201 bp
HGF-R: Forward Reverse	5'-GTTTACTTGTTGCAAGGGAGAAGACT-3' 5'-TAGGGTGCCAGCATTTTAGCA-3'	94°C, 15s	58°C, 60s	40	88 bp
KGF-R: Forward Reverse	5'-TTAAGCAGGAGCATCGCATTG-3' 5'-AACATCCAGGTGGTACGTGTGAT-3'	94°C, 15s	60°C, 60s	40	151 bp
Ubiquitin-c: Forward Reverse	5'-CACTTGGTCCTGCGCTTGA-3' 5'-TTTTTTGGGAATGCAACAACTTT-3'	94°C, 15s	60°C, 60s	40	105 bp

### HGF and KGF concentration in lung homogenates

The proteins HGF and KGF (Quantikine^®^, R&D Systems; respective detection limits were 40 and 15 pg/ml for HGF, KGF) were measured in lung homogenates from 26 out of 35 patients when enough lung sample was available (13 E, 5 S, 8 NS).

### HGF western blotting

Lung homogenates from 4 patients (2 patients with emphysema and 2 patients without emphysema) were examined by Western blotting as previously described [22].

### Statistical analysis

Data were analysed by Statview software (Abacus Concepts, Inc.) and displayed as mean ± SD. Between-group differences were first assessed by non-parametric analysis of variance (Kruskal-Wallis test). In the case of global significant difference, between two groups comparisons were assessed by the non-parametric Mann-Whitney U-test. Correlations were assessed by the Spearman's rank order test. Categorical data were analysed using the Chi-squared test. A p value < 0.05 was regarded as significant.

## Results

### Lung expression of KGF and KGF receptor

**Figure 1:**
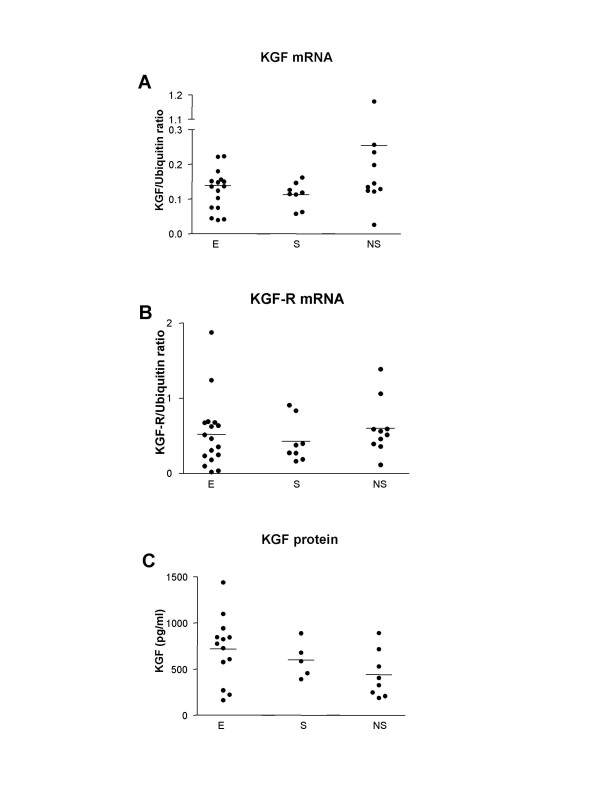
**Expression of KGF (A) and KGF-R (B) mRNA in lung tissue from emphysema and non-emphysema patients**. Results are expressed as a ratio to Ubiquitin in arbitrary units. Individual and mean values (bar) are presented. No difference between groups was found. E: emphysema patients; S and NS: non-emphysematous smoker and non-smoker patients. KGF concentrations in lung homogenates were measured by immunoassay (C). The detection limit was 15 pg/ml. The results are displayed per 1 mg of lung tissue.

KGF mRNA (figure 1A) and KGF protein (figure 1C) were detected in the lungs of all patients. No difference was observed according to the presence of emphysema, and the smoking status.

KGF-R mRNA (figure 1B) was detected in the lungs of all patients. A considerable variability was observed in KGF-R transcript levels in patients with or without emphysema. No difference was observed according to the presence of emphysema and the smoking status.

KGF mRNA, KGF protein and KGF-R mRNA did not correlate with age, cumulative tobacco exposure, period since smoking cessation, use of inhaled corticosteroids, pulmonary function tests and arterial blood gases.

### Lung expression of HGF and HGF receptor

**Figure 2:**
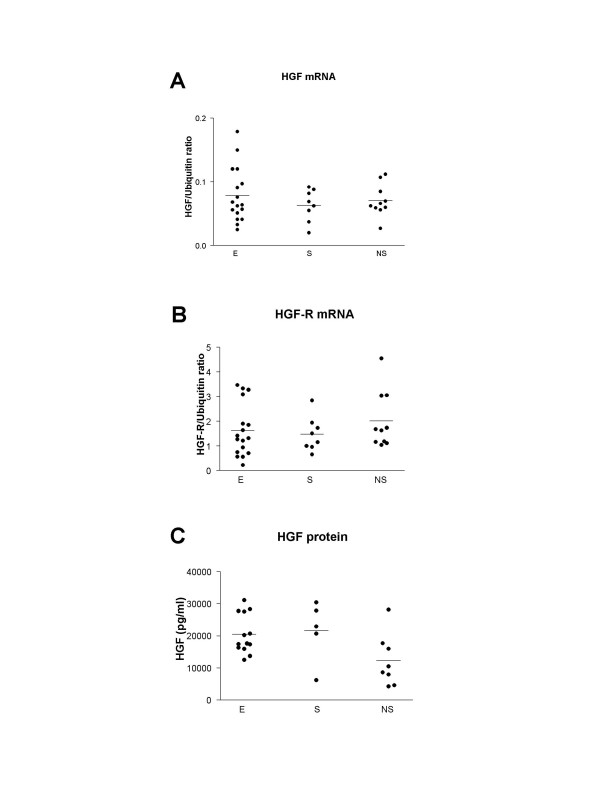
**Expression of HGF (A) and c-met (B) mRNA in lung tissue from emphysema and non-emphysema patients**. Results are expressed as a ratio to Ubiquitin in arbitrary units. Individual and mean values (bar) are presented. No difference between groups was found. E: emphysema patients; S and NS: non-emphysematous smoker and non-smoker patients. HGF concentrations in lung homogenates were measured by immunoassay (C). The detection limit was 40 pg/ml. The results are displayed per 1 mg of lung tissue.

**Figure 3:**
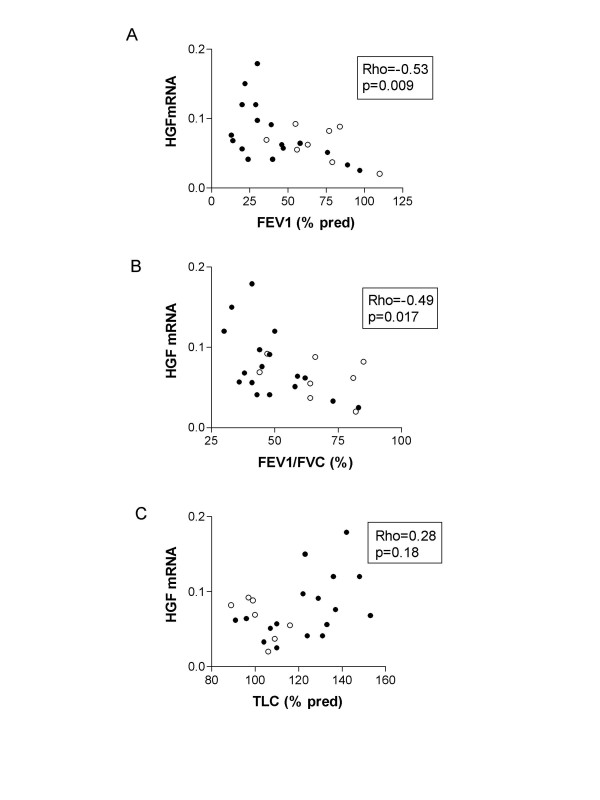
**Correlation between HGF mRNA and pulmonary function in smoker patients**. The ratio of lung HGF to Ubiquitin mRNA was correlated with: (A) forced expiratory volume in one second (FEV1 % predicted), (B) FEV1/FVC (forced vital capacity), but not with (C) total lung capacity (TLC % predicted). Full circle (•): emphysema patients; open circle (o): smoker patients without emphysema.

Although a considerable variability of HGF mRNA expression was observed, no difference was found between emphysema and non-emphysema groups (figure 2A). In emphysema patients, HGF mRNA correlated positively with total lung capacity (TLC) (Rho = 0.51, p = 0.04, n = 17) and negatively with forced expiratory volume in one second (FEV1) (Rho = -0.53, p = 0.03, n = 17) and FEV1/FVC (forced vital capacity) (Rho = -0.54, p = 0.03, n = 17). When all smokers were studied together (smokers with or without emphysema), again, significant correlations between HGF mRNA and FEV1 (Rho = -0.53, p = 0.009, n = 25), and FEV1/FVC (Rho = -0.49, p = 0.017, n = 25) were found. However, the correlation between HGF mRNA and TLC was no more significant (Rho = 0.28, p = 0.18, n = 24) (figure 3). There was no correlation between HGF mRNA and cumulative tobacco exposure.

**Figure 4:**
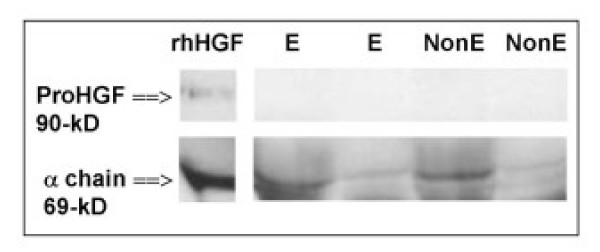
**Western blot analysis of HGF in lung tissue**. Lung homogenates from 4 patients (2 patients with emphysema [E] and 2 non-emphysema patients [NonE]). Recombinant human HGF (R&D Systems: according to the manufacturer, rhHGF is a mixture of proHGF and cleaved mature HGF) was loaded and blotted in parallel. HGF was in the cleaved mature form as evidenced by the detection of the 69-kD α chain and the absence of the 90-kD proHGF form.

HGF protein was detected in lung homogenates from all patients as assessed by immunoassay. HGF concentration was not different between groups (figure 2C). HGF protein correlated positively with residual volume (RV) (Rho = 0.43, p = 0.04, n = 24), TLC (Rho = 0.45, p = 0.03, n = 24) and negatively with FEV1/FVC (Rho = -0.45, p = 0.03, n = 24). Although, no difference of HGF protein concentrations was found between patients' groups, a significant correlation between HGF protein concentrations and cumulative tobacco exposure was observed (Rho = 0.49, p = 0.01, n = 26). As HGF immunoassay measured both proHGF and mature HGF, a western blot analysis was performed to characterize which form of HGF was present in lung homogenates. Western blot (figure 4) demonstrated that HGF was present mainly in the cleaved mature form (presence of the 69-kD alpha chain) both in the non-emphysematous and the emphysematous lungs.

HGF-R mRNA was detected in all patients. We found no difference between groups (figure 2B). Strong correlations were observed between HGF-R mRNA and KGF-R mRNA (Rho = 0.82, p < 0.0001, n = 35) and between HGF mRNA and KGF mRNA (Rho = 0.61, p = 0.004, n = 35) when all patients were taken together.

## Discussion

The involvement of KGF and HGF in lung repair has been widely documented. Numerous studies in vitro and in vivo have demonstrated that KGF and HGF have protective effects in experimental lung injury [15,23]. To our knowledge, this is the first study of KGF and HGF lung expression in human emphysema. Proteases, oxidant injury [2], chronic inflammation [3,5] and apoptosis [6-8] all contribute to the excessive alveolar wall destruction observed in lung emphysema. We hypothezised that a defect of the lung repair process might be associated with the pathophysiology of lung emphysema. Our results show that lung KGF mRNA and KGF protein are not altered in emphysema. In bleomycin-induced lung injury in rats, KGF and HGF increase in the lung [24]. A few clinical studies have assessed KGF concentrations in acute lung injury. Verghese et al reported that KGF was not increased in lung edema fluid whereas HGF was increased and associated with higher mortality [25]. Stern et al reported that KGF and HGF were increased in bronchoalveolar lavage fluid in acute respiratory distress syndrome and associated with a poor prognosis [13]. Recently, Danan et al observed the highest KGF concentrations in tracheal aspirates from premature infants who survived without bronchopulmonary dysplasia, leading to the conclusion that KGF may prevent injury to lung epithelium and enhanceits repair [26].

This study has some methodological limitations. A limited number of patients was studied in each group, especially in non emphysema groups which were mostly composed of lung biopsies obtained at a site distant from localised carcinoma. Furthermore, the patients could only be evaluated at one time point in the course of their disease. Inclusion of smokers without emphysema allowed the differentiation of emphysema-related and tobacco-related events. Because only one tissue sample from surgically resected material was available for examination, the expression of HGF, KGF and their receptors reflects regional disease activity and may be unrepresentative of the entire lung. Indeed, it is well known that emphysema affects different lung regions to a varying extent. Moreover, we evaluated HGF and KGF in lung homogenates only. Future studies should address the expression of HGF and KGF in a more cell-specific fashion.

In our study, although HGF mRNA lung expression was similar in emphysema and non emphysema patients, a correlation was found between HGF mRNA and the deterioration of pulmonary function tests in emphysema patients. The correlation between airflow obstruction and HGF mRNA level was similarly observed when all smokers with or without emphysema were studied, suggesting that emphysema was not a main determinant of HGF mRNA level in the lung. This strong correlation between airflow obstruction and HGF mRNA in smokers suggests that the increase of HGF mRNA was not related to the presence of emphysema but rather to the degree of airflow obstruction. This observation is supported by the correlation between HGF protein in lung homogenates and the FEV1/FVC ratio in our population. These results are in agreement with the observations of Sauleda et al, who reported that HGF protein concentrations were increased in broncho-alveolar lavage of patients with chronic obstructive pulmonary disease as compared to smokers and non-smoker controls [27]. Interestingly, the increased lung expression of other growth factors (fibroblast growth factors 1 and 2 and their receptors) has already been reported in chronic obstructive pulmonary disease [28].

The mechanisms underlying the correlation between airflow obstruction and HGF mRNA in smokers are unclear. Although speculative, we can propose that the mechanical constraints applied to alveolar tissue secondary to airflow obstruction may stimulate HGF production by alveolar epithelial cells, since Yamamoto et al showed that mechanical stretch induced HGF in alveolar type II cells in vitro [29]. Furthermore, airway inflammation could contribute to increase local HGF expression by neutrophils [22] and macrophages [30]. Interestingly, Aharinejad et al have shown that serum HGF concentrations increased at the time of lung graft rejection, a situation associated with airflow obstruction [31].

As HGF and KGF are key factors in the process of alveolar repair [15], we suggest that their production might be not adapted to the degree of alveolar injury. Indeed, in view of the chronic lung injury observed in emphysema, one could expect an increase of HGF and KGF expression as observed in acute lung injury in rats [24] and in humans [25,13]. However the direct assessment of HGF content in lung homogenates is technically difficult. Indeed, HGF is a heparin binding growth factor. High concentrations of inactive precursor of HGF (proHGF) are probably bound to proteoglycans of the extracellular matrix and may not be assayed in the lung homogenates by immunoassay. In this study, western blot analysis showed that HGF was only in the mature active form, both in lung biopsies from emphysema and non-emphysema patients.

Recently, HGF has been shown to stimulate pulmonary regeneration and to improve pulmonary function in animal models of elastase-induced lung emphysema [20,19]. Preserved expression of HGF-R could allow therapeutic use of growth factors in lung emphysema. Further studies are needed to assess the therapeutic potential of HGF and KGF in lung emphysema.

## Conclusion

The main results of our study are that: i) KGF and HGF lung expression is preserved in emphysema patients, ii) HGF-R and KGF-R mRNA are consistently expressed in the lung of emphysema patients and are not modified by the smoking status, iii) HGF mRNA correlates with the severity of airflow obstruction in smokers.

## List of abbreviations

α1-AT: α1-antitrypsin

E: emphysema

FEV1: forced expiratory volume in one second

FGF: fibroblast growth factor

FVC: forced vital capacity

HGF: hepatocyte growth factor

KGF: keratinocyte growth factor

mRNA: messenger ribonucleic acid

NS: non-smoker without emphysema

RT-PCR: reverse transcriptase-real-time polymerase chain reaction

RV: residual volume

S: smoker without emphysema

SD: standard deviation

TLC: total lung capacity

## Competing interests

The author(s) declare that they have no competing interests.

## Authors' contributions

MB and AB equally participated in the design of the study, conducted the majority of the research experiments and drafted the manuscript.

VL participated in the majority of the research experiments.

MF participated in the design of the study.

JM and PS conducted some experiments.

GL participated in the design of the study.

MD and BC conceived of the study, participated in its design, and in drafting the manuscript.

All authors read and approved the final version of the manuscript.

## Pre-publication history

The pre-publication history for this paper can be accessed here:


